# Current Status of Internal Cardioversion in Atrial Fibrillation

**Published:** 2002-04-01

**Authors:** Andreas Plewan, Eckhard Alt

**Affiliations:** Medizinische Klinik, Klinikum rechts der Isar, Technische Universität Munchen, Germany

**Keywords:** atrial fibrillation, internal cardioversion

 For more than 30 years transthoracic external cardioversion has been an established method for the restoration of sinus rhythm in patients with persistent atrial fibrillation. It was first described by Lown in 1963 [1]. Though the success rate for external cardioversion ranges from 60-90 % [2,4], there is reduced efficacy in those patients with a high body-mass index and an increased transthoracic diameter [5].

The method of internal cardioversion for restoration of sinus rhythm using transvenous electrodes has been reported in several animal [6,7] and human studies [8-15]. Cooper et al [6] tested multiple electrode configurations in a sheep model of atrial fibrillation. They demonstrated that the optimal single current pathway for internal atrial defibrillation employed two electrodes that surrounded both atria (e.g., right atrial appendage and distal coronary sinus). Similar results have been reported in several human studies [12-14]. Internal cardioversion has been shown to be superior to conventional external cardioversion in terms of primary success rate, energy requirements and the need for sedation; this superiority holds especially true for patients with a high body mass index of > 25 kg/m2 and increased transthoracic diameter [1]

The initial human data has been collected using two separate catheter for internal cardioversion. Though this approach demonstrated some advantages, there are also clear disadvantages, such as lack of ventricular backup stimulation in cases of post shock bradycardia, prolonged fluoroscopy time and the need for venous access via primarily the lower limb. These disadvantages increase the risk of bleeding complications, especially in patients with anticoagulation.

Recently published data have demonstrated the benefit of a single lead catheter with two shock arrays on a balloon guided pulmonary artery catheter, when compared to the previous two catheter technique [12]. Optional ventricular backup pacing, preferable venous access via the upper limb, reduced procedural and fluoroscopy time are the major advantages. This new balloon-tipped cardioversion catheter (Figure 1 and 2) resembles a regular Swan-Ganz catheter. A guide-wire introduced via a central lumen allows facilitated searing and positioning of this device. The central lumen also provides means to draw blood samples, infuse drugs or perform haemodynamic measurements (pulmonary artery and wedge pressure). The proximal and distal high-energy electrode arrays for internal defibrillation consist of six 0.5 cm long platinum rings with a total surface area of 2.4 cm2 for each array. The middle ring of the proximal array is connected individually for atrial sensing and pacing, while the others are connected in parallel (atrial array). One pole located in the right ventricular outflow tract - between both arrays - serves for ventricular pacing and sensing. The six distal pulmonary rings are connected in parallel for internal cadioversion shock delivery.

For cardioversion with this new device an electrode configuration in the right atrium and left pulmonary artery is suggested. A randomized trial could demonstrate that this configuration provides a homogeneous electrical field for effective cardioversion, and slightly higher energy requirements compared to a lead position in the right atrium and distal coronary sinus [12], as described earlier. However the use of single lead catheters with a shock array position proximal in the right atrium and distal in the left pulmonary artery has contributed to facilitate the procedure of internal cardioversion of atrial fibrillation considerably.

Ventricular backup pacing is an important safety tool, not only for a subgroup of patients with post shock bradycardia, but also for bradycardia after the administration of antiarrhythmic drugs or beta-blockers. The possibility of post shock atrial pacing also offers new therapeutic options in preventing the early recurrence of atrial fibrillation. Immediately after cardioversion 60% of all patients develop premature atrial contractions [16]. Recently published data suggest that short coupling intervals of the premature atrial beat predict an early relapse of atrial fibrillation [16]. Heterogeneity of refractoriness and conduction give way for a premature stimulus to reinduce atrial fibrillation. Immediately after cardioversion, electrical remodeling has shortened the refractory period of the atria, thereby facilitating relapse [16,17]. Atrial overdrive stimulation after successful internal cardioversion for the prevention of atrial premature beats promotes the homogeneity of the atrial wavefront. Atrial stimulation at higher rates therefore can prevent early recurrence of atrial fibrillation, thereby contributing to the clinical efficacy of internal cardioversion [18].

 For internal cardioversion of atrial fibrillation an electrode position with the distal tip in the left pulmonary artery is currently used preferably (Figure 1). Due to the manufactured curvature of the Swan-Ganz type catheter, this catheter has the tendency to favor the right pulmonary artery when advanced normally. Therefore the use of a lead configuration in the right atrium and right pulmonary artery would facilitate the procedure of internal cardioversion (Figure 2). A recently finished study at the Rechts der Isar Medical Center in Munich revealed that the efficacy of internal cardioversion of atrial fibrillation with the distal shock array in the right pulmonary artery is 88 %. This success rate is somewhat lower than for a positioning in the left pulmonary artery. The reason for this difference might only be anticipated. The creation of a homogenous electrical field with sufficient strength is mandatory for conversion of ventricular fibrillation [13]. The same may hold true for AF. With respect to homogeneity of field strength, one would assume that direct application of the energy to the two areas within or surrounding the heart should result in increased myocardial field strength and decreased energy loss. While an electrode position in the right atrium and left pulmonary artery creates an electric field vector encompassing large areas of both atria, an electrode position right atrium and right pulmonary artery does not include parts of the left lateral atrium. This exclusion results in a loss of homogeneity of the electric field strength and therefore mandates slightly higher cardioversion energies. Nevertheless, the success rates for internal cardioversion with the distal tip in the right pulmonary artery is comparable to the efficacy of external cardioversion. This lead position furthermore facilitates the internal cardioversion procedure, because the positioning of the single lead balloon tipped cardioversion catheter in the right pulmonary artery is comparable to the positioning of a regular Swan-Ganz catheter.

Though the design of the cardioversion catheter is similar to a regular Swan-Ganz balloon catheter, the cardioversion catheter offers the additional facilities of cardioversion, cardiac pacing and, in the newest generation of these electrode systems even haemodynamic measurements by means of thermodilution. These qualities render the cardioversion catheter suited for application in the electrophysiological laboratory as well as in emergency and intensive care settings. More than 30 % of patients undergoing cardiovascular surgery develop persistent atrial fibrillation [20]. In these settings, the new device combines not only routine haemodynamic monitoring, identical to a regular Swan-Ganz catheter, but also offers means for effectively cardioverting atrial fibrillation without the assistance of an anesthesiologist, a widespread prerequisite for external cardioversion procedures in outpatient departments. The balloon guided single lead cardioversion catheter offers a valid alternative to external cardioversion, and can be operated by non-cardiologist as well. Currently ongoing studies assess the value of the combined Swan-Ganz thermodilution and cardioversion-catheter in the postoperative ICU-setting.

## Figures and Tables

**Figure 1:**
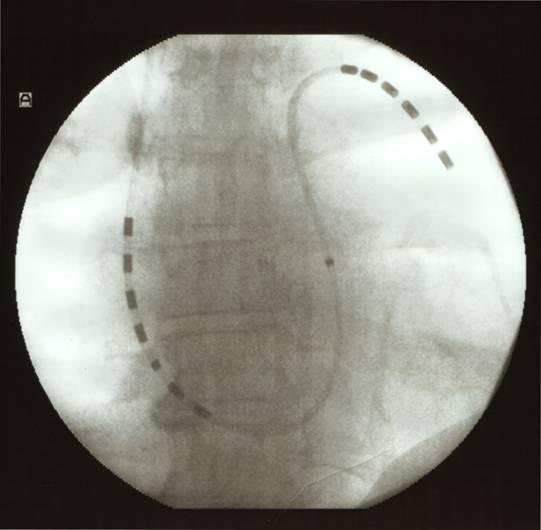
Single lead ballon guided cardioversion catheter introduced via the right cubital vein. The proximal shock array contacts the basal right atrial wall and the distal shock array is positioned in the left pulmonary artery

**Figure 2:**
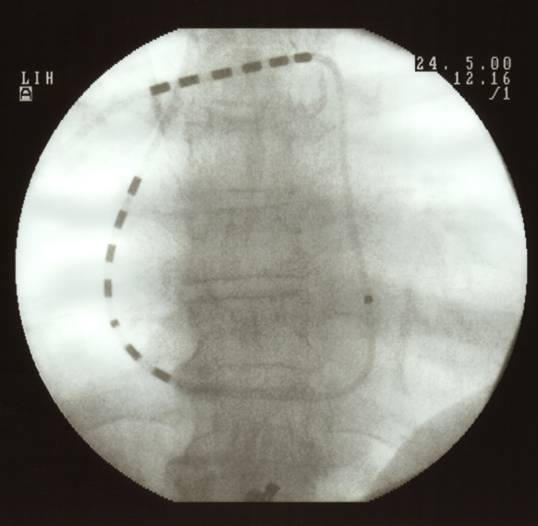
Same patient as in figure 1. The distal shock array is in the right pulmonary artery in a position similar to a regular Swan Ganz catheter for haemodynamic monitoring
